# Antibacterial activity of poly-l-arginine under different conditions

**Published:** 2017-04

**Authors:** Mohaddeseh Sepahi, Razieh Jalal, Mansour Mashreghi

**Affiliations:** 1 Department of Chemistry, Faculty of Sciences, Ferdowsi University of Mashhad, Mashhad, Iran; 2 Cell and Molecular Biotechnology Research Group, Institute of Biotechnology, Ferdowsi University of Mashhad, Mashhad, Iran; 3 Department of Biology, Faculty of Sciences, Ferdowsi University of Mashhad, Mashhad, Iran

**Keywords:** Antibacterial activity, *Escherichia coli* O157:H7, Glycine, Poly-l-arginine, *Staphylococcus aureus*

## Abstract

**Background and Objectives:**

Arginine-rich peptides are an important class of antimicrobial peptides (AMPs) that exert their antibacterial activity via a lytic mechanism. Although the antibacterial activity of arginine-rich peptides has been already evaluated, no reports have so far been evaluated the influence of reaction conditions on their antimicrobial potential. The aim of the present study was to investigate the influence of pH, temperature, and glycine on antibacterial activity of poly-l-arginine (PLA) with a molecular weight of 5–15 kDa against *Escherichia coli* O157:H7 and *Staphylococcus aureus.*

**Materials and Methods:**

The percentage of growth inhibition of PLA against both bacteria was analyzed at various pH, temperatures and sub-inhibitory concentrations of glycine by two-fold broth microdilution method.

**Results:**

The results showed that PLA had antibacterial activity against *E. coli* O157:H7 and *S. aureus* and the inhibitory effect increased with increasing PLA concentration. The antimicrobial activity of PLA against both microorganisms was higher in basic media than under acidic or neutral conditions. At 1/2 times the MIC, heat treatment intensified the toxicity of PLA against *E. coli* O157:H7 whereas the susceptibility to PLA seems to be temperature independent for *S. aureus*. The MICs of glycine against *E. coli* O157:H7 and *S. aureus* were 12.5 and 25 mg ml^−1^, respectively. The antibacterial activity of PLA against both microorganisms increased, as indicated by the increasing growth inhibition percentage of this peptide with increasing glycine concentration.

**Conclusion:**

The antibacterial activity of PLA against *S. aureus* and *E. coli* O157:H7 depends on pH and glycine concentration.

## INTRODUCTION

Food safety is a worldwide major public health concern and research on controlling foodborne pathogens is important for food safety. Although various methods are applied to eliminate or control pathogenic microorganisms in the food industry, avoiding microbial contamination is still difficult (1). Basic polyamino acids have been reported to have broad-spectrum activity against Gram positive and Gram negative bacteria, fungi, viruses, and eukaryotic parasites (2, 3). Lysine- and arginine-rich proteins are reported to possess antimicrobial activity, inhibit tumor growth, and enhance drug delivery across biological membranes and tissues (4–7). Basic homopoly(amino acid)s and homooligo(amino acid)s, such as poly-l-lysine (PLL) and poly-l-arginine (PLA), have attracted considerable attention due to not only their antibacterial activity but also their non-toxicity and excellent biocompatibility (7, 8).

L-Arginine is a semi-essential amino acid and widely used as nutritional supplement in food and beverage industries to provide arginine and as adjuvant therapy for cardiovascular and neuronal disease (9, 10). Since the guanidinium group of arginine can form extensive hydrogen-bond and ionic interactions, PLA found to have greater ability to enhance membrane permeability compared to PLL (11–13). Several suggested applications for PLA include drug delivery carriers (14), anticancer vaccines (15), gene expression (5, 16), neuroprotective agent (10), and polyelectrolyte film formation (17). PLA is a polyelectrolyte with an electrolyte group on every repeat unit which its charge depends on the properties of the solution, such as pH, temperature, and salt concentration (8); thus its effectiveness can be a function of the solution conditions. To promote the application of PLA, elucidating the impact of environmental factors on its antibacterial activity is particularly important.

The objective of the present study was to investigate antibacterial activity of PLA and gather information about factors that may influence its antibacterial activity. *E. coli* O157:H7 and *S. aureus*, two of the major foodborne pathogens, were chosen as model food systems to evaluate antibacterial activity of PLA under various conditions, including alteration of the media pH and treatment temperature and the addition of sub-inhibitory concentration of glycine to the media.

## METHODS

PLA with molecular weight ranging from 5,000 to 15,000 Da was purchased from Sigma (Steinheim, Germany). Glycine and nutrient broth (NB) medium were from Merck (Darmstadt, Germany). *E. coli* O157:H7 (NTCC: 12900) and *S. aureus* (ATCC: 25923), two pathogenic foodborne bacteria, were used as Gram-negative and Gram-positive bacterial models, respectively.

### Bacterial culture conditions and antibacterial test.

The antibacterial activity of PLA was analyzed by the broth microdilution method in a microplate. Serial two-fold dilutions of PLA (1.95–31.25 μg ml ^−1^) were prepared in NB medium. A suspension of exponentially growing bacteria was added to each medium to attain a final bacterial concentration of 10 ^6^ CFU (colony forming unit) ml ^−1^ . Samples were placed in 96-well microtiter plates and incubated for 24 hours at 37°C on a reciprocal shaker (120 rpm). Bacterial growth was monitored every 2 h by measuring the optical density value at 630 nm (OD _630 _). Inoculated NB culture medium without the PLA solution was included as positive control and uninoculated NB medium as negative control for each assay. The lowest concentration of PLA that inhibited growth after 24 h incubation was obtained as the minimum inhibitory concentration (MIC) of PLA against the test bacteria. Also, the percentage of growth inhibition (GI %) for both microorganisms in comparison with positive controls was determined using Eq. 1:

$$\text{GI}\%=\frac{{\mathrm{OD}}_{630}\;\mathrm{at}\;\mathrm{the}\;\mathrm{presence}\;\mathrm{of}\;\mathrm{antibacterial}\;\mathrm{agent}\;(s)}{{\mathrm{OD}}_{630}\;\mathrm{of}\;\mathrm{positive}\;\mathrm{control}}\times 100(1)$$

### Influence of pH and temperature on the antibacterial activity of PLA.

The experiments were conducted at pH and temperature range in which bacterial cells could grow normally. Antibacterial tests were performed at pH range of 5–10 for *E. coli* O157:H7 and 5–9 for *S. aureus* and different incubation temperatures (25–42°C). Bacterial suspensions with different concentrations of PLA (3.90 – 15.60 μg ml ^−1^ for *E. coli* O157:H7; 1.95–62.50 μg ml ^−1^ for *S. aureus*) at mentioned pH and temperature values were incubated for 24 h. The bacterial growth curves were determined by optical density value analysis. It is clear that some harsh condition reactions such as acidic or alkaline pH and heat temperature would reduce the growth of bacterial cells. Thus in order to exclude the inhibitory effect of reaction conditions on the bacterial growth, bacterial cultures without PLA at the same pH and temperature conditions of the treated bacterial cultures were used as positive controls. Therefore, each treated bacterial culture had its own positive control and the growth of treated groups was compared to those of positive control groups. Thus, all the observed antibacterial effect is referred to the antibacterial activity of PLA and not to reaction conditions of the experiments. Also, uninoculated NB medium containing the same concentration of PLA was incubated under the same conditions of pH and temperature and served as blank control. The MIC and GI % of each treatment at various reaction conditions was calculated in comparison with its own positive control using Eq. 1.

### Influence of glycine on the antibacterial activity of PLA.

Glycine is the simplest amino acid and it is demonstrated that an excess amount of glycine can inhibit the growth of many bacteria (18, 19). For testing the susceptibilities of *E. coli* O157:H7 and *S. aureus* to glycine, cells (10 ^6^ CFU ml ^−1^) were incubated at 37°C for 24 h in NB (pH 7) containing different concentrations of glycine (3.12 – 25 mg ml ^−1^) and the MIC value was determined. A bacterial culture without glycine was served as positive control. To investigate the influence of glycine on the antibacterial activity of PLA against both microorganisms, the sub-inhibitory concentrations of PLA and glycine (1/2 × MIC and 1/4 × MIC) alone or in combination were prepared in NB (pH 7) inoculated with tested strains (10 ^6^ CFU ml ^−1^) and incubated for 24 h at 37°C in a rotatory shaker at 120 rpm. Bacterial growth was monitored every 2 h by measuring the OD _630_ . Positive and negative controls were also used. The GI % of PLA and glycine alone or in combination was determined using Eq. 1.

### Statistical analysis.

The statistical analyses were carried out using Minitab 16 software. The level of statistical significance was chosen to be 0.05. All the experiments were performed triplicate and results are presented as mean±standard deviation (SD).

## RESULTS

### Influence of pH and temperature on the growth of *E. coli* O157:H7 and *S. aureus*.

When *E. coli* O157: H7 was cultured in NB medium at different initial pH ranging from 5 to 11, the maximum growth was observed at pH 7 for temperatures of 25, 37, and 42°C and the microorganism was not able to grow at pH 11 and 42°C. For *S. aureus*, the maximum growth at 37°C was occurred at pH 8 and the strain failed to grow at pH 9 and above. At temperatures 25 and 42°C, the most favorable pH for the microorganism to achieve the maximum growth was 7. *S. aureus* was not able to grow at pH 8–11 and 42°C whereas at 25°C no growth was observed at pH 10 and 11.

### Antimicrobial activity of PLA against *E. coli* O157:H7 and *S. aureus*.

The MIC values of PLA against *E. coli* O157:H7 and *S. aureus* at different pH values and temperatures are shown in Table 1. PLA showed to be more active on *S. aureus* than *E. coli* O157:H7 at pH 7 and 37°C which is in agreement with Conte et al (20). Different values of MIC against *E. coli* O157: H7 and *S. aureus* may be related to their different cell wall structure (21).

**Table 1.** Minimum inhibitory concentrations (μg ml^−1^) of PLA against *E. coli* O157:H7 and *S. aureus* at various pH values and temperatures.

**Incubation Temperature (°C)**	**Strains**	**pH**								

		**5**	**6**	**7**	**8**	**9**	**10**	**11**
**25**	*E. coli* O157:H7	15.60	15.60	15.60	7.80	7.80	7.80	7.80
	*S. aureus*	> 62.50	31.20	7.80	3.90	3.90	-	-
**37**	*E. coli* O157:H7	15.60	15.60	15.60	15.60	7.80	7.80	7.80
	*S. aureus*	31.20	31.20	3.90	3.90	-	-	-
**42**	*E. coli* O157:H7	15.60	15.60	7.80	7.80	7.80	7.80	-
	*S. aureus*	15.60	15.60	7.80	-	-	-	-

### Influence of pH on the antibacterial activity of PLA.

**Fig. 1.**
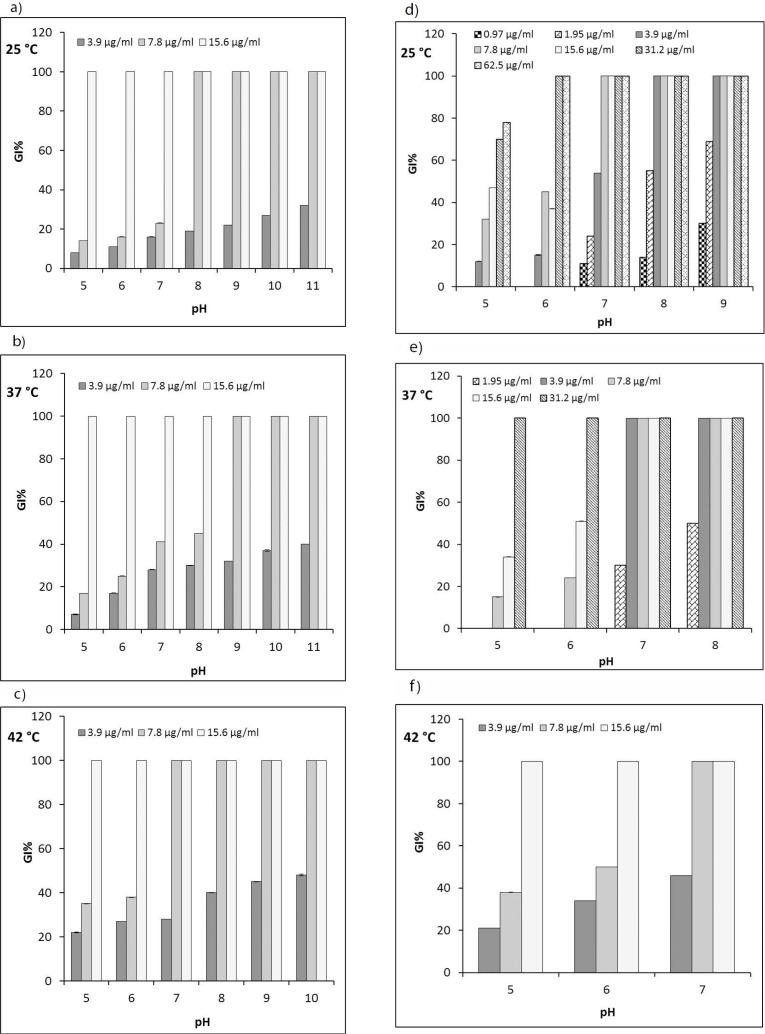
The influence of pH and temperature on the antibacterial activity of poly-l-arginine (PLA). The percentage of growth inhibition (GI%) by PLA against *E. coli* O157:H7 (a, b, and c) and *S. aureus* (d, e, and f) at various PLA concentrations, pH values, and temperatures after 24 h

The results showed that the overall influence of pH on the antibacterial activity of PLA against both bacteria was similar. According to the MIC values at different pH values and temperatures, a wider range of PLA concentrations was used to explore its antibacterial activity against *S. aureus* (1.95–62.50 μg ml ^−1^) in comparison with *E. coli* O157: H7 (3.90–15.60 μg ml ^−1^). For all examined temperatures, MIC values of PLA decreased as the pH increased from acidic to alkaline (Table 1). At concentrations below the MICs, the bacterial growth of both microorganisms decreased as the concentration of PLA and pH increased (Fig. 1). It suggests a pH-dependent mechanism for PLA antimicrobial properties.

### Influence of temperature on the antibacterial activity of PLA.

The influence of temperature on the antibacterial activity of PLA against *E. coli* O157:H7 is shown in Fig. 1a, 1b, and 1c. Heat treatment increased the percentage of growth inhibition at concentrations below the MICs. The MIC of PLA against *E. coli* O157:H7 increased by enhancing temperature from 25 to 37°C only at pH 8, as tested in the pH range of 5–11 (Table 1). Also, the MIC value at pH 7 decreased when the treatment temperature was increased from 37 to 42°C. As shown in Table 1, the MIC at pH 7 against *S. aureus* was higher at 25°C than at 37°C. At these temperatures, *S. aureus* showed a relatively wide range of MIC values (3.9 to > 62.5 μg ml ^−1^) compared with *E. coli* O157:H7 (7.8 to 15.6 μg ml ^−1^). At acidic pH, especially at pH 5, the MIC values decreased when the treatment temperature was increased. At concentration of 7.8 μg ml ^−1^ and at acidic pH (5 and 6), the GI% of PLA was also lower at 37°C than at 25°C (Fig. 1d and 1e). As evident in Fig. 1, the stronger inhibitory influence of PLA at alkaline pH was also observed for all temperatures (25–42°C).

### Effect of process parameters on the antibacterial activity of PLA.

The response surface design using statistical software (Minitab 16) was used to pick factors that influence on the antibacterial activity of PLA (22). Of the three analyzed factors, pH (p = 0.01) and the concentration of PLA (p = 0.006) were significant to the antibacterial activity of PLA against *S. aureus.* Interaction between pH and PLA concentration was also significant as shown by low p value (p = 0.049). It was observed that PLA concentration was the only significant factor that affected its antibacterial activity on *E. coli* O157:H7 (p < 0.05).

### Influence of glycine on the antibacterial activity of PLA.

**Fig. 2.**
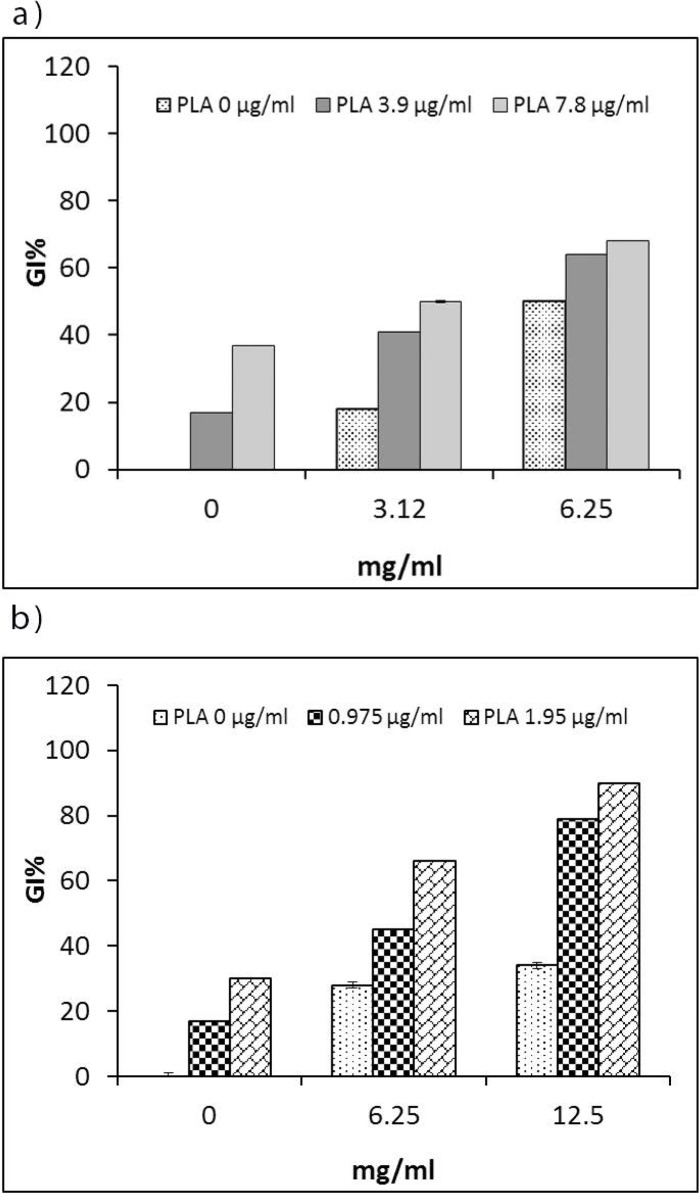
The influence of glycine on the antibacterial activity of poly-l-arginine (PLA). The percentage of growth inhibition (GI%) by PLA against *E. coli* O157:H7 (a) and *S. aureus* (b) in the presence of sub-inhibitory concentrations of glycine (1/2 and 1/4 × MIC; mg ml^−1^) after 24 h

Antibacterial properties of glycine were evaluated against *E. coli* O157:H7 and *S. aureus* by the microdilution method at 37°C and pH 7. Our results showed that the growth of both strains was decreased by glycine in a concentration dependent manner. The MICs of glycine against *E. coli* O157:H7 and *S. aureus* were 12.5 and 25 mg ml ^−1^ , respectively. Furthermore, glycine at concentration of 6.25 mg ml ^−1^ resulted in 28 and 50% reductions, respectively, in growth of *S. aureus* and *E. coli* O157:H7. Since the cell wall in gram-negative bacteria is thinner than in gram-positive bacteria, the required amount of glycine to suppress *E. coli* O157:H7 proliferation is lower than that required to suppress *S. aureus* (23). To evaluate the influence of glycine on the antibacterial activity of PLA, bacterial cells were treated simultaneously with different sub-inhibitory concentrations of PLA and glycine. The antibacterial activity of PLA increased in the presence of glycine. As shown in Fig. 2, the range of fold-increase in antibacterial activity of PLA (judged by GI%) against *E. coli* O157:H7 and *S. aureus* in the presence of glycine with respect to its absence were 1.3- to 3.8-fold and 2.2- to 4.6-fold, respectively.

## DISCUSSION

Arg, as an amino acid, found to play important roles in binding, disrupting, and permeabilizing of lipid membranes. The use of basic homopolyamino acids, such as poly-l-arginine (PLA), is a promising strategy for developing antibacterial agents relevant to the food industry. Since the positively charged peptides with both hydrophobic and hydrophilic sides are soluble in aqueous environments and can enter lipid-rich membranes (24), they are able to pass through the bacteria’s outer membrane and either depolarize the cytoplasmic membrane and cause cell lysis or pass through the cytoplasmic membrane to interact with the inner cellular contents (25–27). Conte et al. (2007) demonstrated the antibacterial activity of various arginine- and lysine-rich polycationic proteins on a range of foodborne pathogens. They showed that α-PLA can pass through cytoplasmic membranes and entry into the cytoplasm of bacterial cells (20).

To the best of our knowledge, this is the first report on the influence of pH on the antibacterial activity of PLA. The impacts of pH on cationic AMPs activity can via either affecting the electrostatic interactions involved in the initial binding step or a change in the ionic strength of the solution (28). The net charge of the target molecules on the bacteria or of the cationic AMP, depending on the pKas of the functional groups involved, may change as the pH changes. Also, the change in the relative concentrations of the various charged species of the buffer may occur with a change in pH. Mackay et al. (1984) showed that the histidine-rich antimicrobial peptides have greater antibacterial activity at low pH than at a neutral pH (29). The antibacterial activity of clavanins and histidine-rich antimicrobial peptides were found to be substantially greater at low pH (pH 5.5) than at a neutral pH (pH 7.4) while clavanin AK, a synthetic variant of clavanin A acid containing 4 histidine to lysine substitutions, was active at both pH 5.5 and pH 7.4 (1). Histidine residues have a pKa near 6.5, so histidine-rich peptides have high net positive charges at acidic pH and are relatively uncharged at neutral pH (1). Here, PLA is found to kill *S. aureus* and *E. coli* O157:H7 much better at higher pH than lower pH. The likely explanation for the observed pH behavior is as follows. *S. aureus* is known to have large amounts of anionic teichoic acids (TAs) in its thick peptidoglycan (PG) layer (30, 31) and the ribitol groups of TAs are esterified to D-Ala residues containing free amino groups (31, 32). At low pH, the net negative charge on the PG layer of *S. aureus* is reduced because of the positive charges from the amino groups of D-Ala (pKa 8.4) and this layer becomes neutral or even slightly positively charged. Although, PLA (pKa near 12 for the isolated amino acids) would remain positively charged at low pH values, thus it has lowered attraction to, or be repelled from, the PG layer. At high pH, the anionic character of the PG layer is increased since some D-alanyl esters in TAs are hydrolyzed and also the amino groups of the D-Ala become deprotonated (32, 33). Thus, PLA may bind and pass through the PG layer to kill with a low MIC value at high pH. The pH behavior observed with *E. coli* O157:H7 may be due to the presence of free-amino containing groups, such as ethanolamine and glucoseamine, in its anionic LPS layer which exhibit pH behavior similar to the D-Ala residues in *S. aureus* (34, 35).

Our results indicated that the antibacterial activity of PLA was temperature-dependent at subinhibitory concentrations (sub-MICs) for *E. coli* O157:H7. Previous studied had shown that the LPS O-polysaccha-rides and the modifications of the lipid A with aminoarabinose, phosphoethanolamine, and palmitate were linked to resistance to antimicrobial peptides in some bacteria (36–38). The increased antibacterial effect of PLA at higher temperatures could be attributed to the temperature-regulated LPS structural changes and lipid A modifications. The differences in the fatty acid profiles and fluidity of bacterial cell membrane could be possible explanations for the reduced antibacterial activity found at lower temperatures (39, 40). Here, *S. aureus* was able to grow at pH 5–9 for temperatures 25°C and 37°C, while at 42°C its growth was only observed at pH levels 7 and lower. Therefore, we compared the influence of temperature (25, 37, and 42°C) on the antibacterial activity of PLA against *S. aureus* at pH 5, 6, and 7. The antibacterial activity of PLA seems to be temperature-independent at subinhibitory concentrations (sub-MICs) for *S. aureus*. PLA antibacterial activity against *S. aureus* and *E. coli* O157:H7 were decreased at acidic pH and pH 8, respectively, as increasing temperature from 25 to 37°C. The increased PLA antibacterial activity found at lower temperatures (25°C) may be due to alterations in the energy-dependent active transport mechanisms in bacterial membranes or a temperature dependent mode of action at the bacterial ribosome (41). These theoretical explanations require further study by experimental data.

To the best of our knowledge, the effect of glycine against *S. aureus* and *E. coli* O157:H7 was not studied before. An excess of glycine, the simplest amino acid, is known to inhibit the growth of many bacteria and has low level of toxicity in animals. Several mechanisms of antimicrobial activity of glycine have been postulated in the past. Glycine may inhibit a number of available active enzymes, such as UDP-*N*-acetylmura-mate-alanine ligase, DD- and DL- carboxypeptidases, which are important for the formation of cell wall and peptidoglycan (42, 43). Also, glycine may weaken the structure of the cell wall by acting on the replacement of L-alanine residues as an analogue of either L- or D-alanine (44). Minami et al. (2004) showed that glycine has antibacterial activity against *Helicobacter pylori in* a concentration-dependent manner and increases the inhibitory effect of amoxicillin (19). Glycine betaine (N,N,N-trimethylglycine) analogues have reported to exert a toxic activity against several bacterial strains belonging to diverse taxonomic groups (45).

Several reports have been demonstrated that functionalization of some compounds with amino acids leads to increased their activity. For example, copper and cobalt amino acids complexes have been reported to exhibit antibacterial activity against Gram- positive and Gram-negative bacteria (46). The functionalization of multi-walled carbon nanotubes (MWCNTs) with cationic amino acids were also proven to be effective against *E. coli* and the resistant strains of *S. aureus* due to electrostatic adsorption of positive charges of the functional groups on MWCNTs surface with bacteria membrane (27). Al-Maythalony et al. showed that (His)Cd(SeCN) _2_ and (Gly)Cd(SeCN) _2_ complexes have antibacterial activity compared to Cd(SeCN) _2_ , while (His)Hg(SeCN) _2_ and (Gly)Hg(SeCN) _2_ complexes have no significant antibacterial activity (47). In the present study, as far as we know, we report for the first time the influence of glycine on the antibacterial activity of PLA.

In conclusion, this study demonstrated that PLA has antibacterial activity against *S. aureus* and *E. coli* O157:H7 and its efficiency is sensitive to the change in pH. Also, this study showed that glycine may be a useful antimicrobial agent, alone or in combination with PLA, against both microorganisms. To gain more insight into the biological role of PLA and glycine, testing the activities against pathogens *in vivo* is recommended. Moreover, further investigations are required to understand the mechanism (s) and factors involved in the antibacterial efficiency of PLA.
